# Repression of RNA Polymerase II Elongation *In Vivo* Is Critically Dependent on the C-Terminus of Spt5

**DOI:** 10.1371/journal.pone.0006918

**Published:** 2009-09-09

**Authors:** Hui Chen, Xavier Contreras, Yuki Yamaguchi, Hiroshi Handa, B. Matija Peterlin, Su Guo

**Affiliations:** 1 Department of Biopharmaceutical Sciences, and Programs in Biological Sciences and Human Genetics, University of California San Francisco, San Francisco, California, United States of America; 2 Departments of Medicine, Microbiology and Immunology, Rosalind Russell Medical Research Center, University of California San Francisco, San Francisco, California, United States of America; 3 Graduate School of Bioscience and Biotechnology, Tokyo Institute of Technology, Yokohama, Japan; Institute of Genetics and Molecular and Cellular Biology, France

## Abstract

The stalling of RNA polymerase II (RNAPII) at the promoters of many genes, including developmental regulators, stress-responsive genes, and *HIVLTR*, suggests transcription elongation as a critical regulatory step in addition to initiation. Spt5, the large subunit of the DRB sensitivity-inducing factor (DSIF), represses or activates RNAPII elongation *in vitro*. How RNAPII elongation is repressed *in vivo* is not well understood. Here we report that CTR1 and CTR2CT, the two repeat-containing regions constituting the C-terminus of Spt5, play a redundant role in repressing RNAPII elongation *in vivo*. First, mis-expression of Spt5 lacking CTR1 or CTR2CT is inconsequential, but mis-expression of Spt5 lacking the entire C-terminus (termed NSpt5) dominantly impairs embryogenesis in zebrafish. Second, NSpt5 de-represses the transcription of *hsp70-4* in zebrafish embryos and *HIVLTR* in cultured human cells, which are repressed at the RNAPII elongation step under non-inducible conditions. Third, NSpt5 directly associates with *hsp70-4* chromatin *in vivo* and increases the occupancy of RNAPII, positive transcription elongation factor b (P-TEFb), histone H3 Lys 4 trimethylation (H3K4Me3), and surprisingly, the negative elongation factor A (NELF-A) at the locus, indicating a direct action of NSpt5 on the elongation repressed locus. Together, these results reveal a dominant activity of NSpt5 to de-repress RNAPII elongation, and suggest that the C-terminus of Spt5 is critical for repressing RNAPII elongation *in vivo*.

## Introduction

The production of mRNA is a multi-step process that involves transcription initiation, elongation, and termination [1]–[3]. For decades, the major mechanism of gene regulation in higher eukaryotes is thought to reside at the level of recruitment of RNAPII by sequence-specific DNA binding factors, despite the presence of RNAPII proximal to the promoters of a few genes including *hsp70* [4] and *HIVLTR* [5] in un-induced conditions. But recently, it has been observed that many transcriptionally repressed genes have promoter-proximal paused or stalled RNAPII [6]–[9]. Moreover, biochemical studies have identified over a dozen proteins and a small nuclear RNA, which regulate RNAPII elongation *in vitro* [2], [10], [11].

Among the identified elongation factors, the DRB-Sensitivity-Inducing-Factor (DSIF) that is composed of Spt4 and Spt5, can repress and activate RNAPII elongation on an artificial DNA template under different assay conditions *in vitro* [12], [13]. Since Spt4 is a small protein and not essential for yeast survival [14], most studies including ours have focused on Spt5. The C-terminal repeat 1 (CTR1) of Spt5 [15]–[19], together with RNAPII C-terminal domain (CTD) [13], [20] and Negative Elongation Factors (NELF) [21] are targets of phosphorylation by P-TEFb, a protein kinase composed of CDK9 and Cyclin T subunits, which reverses elongation repression and promotes positive elongation.

Despite these advancements, how Spt5 regulates RNAPII elongation *in vivo* is not well understood. Although genetic analyses in yeast [22], *C. elegans* [23], *Drosophila* [24], and zebrafish [25]–[27] have revealed an essential role of Spt5 in cell growth and embryonic development, these studies do not address whether these phenotypes are caused by a defect in RNAPII elongation, since Spt5 has also been implicated in regulating mRNA capping [28], [29], splicing [30], 3′ end processing [31], [32], and mRNA export [33]. Despite that point mutations located in the C-terminus of zebrafish [25) or Drosophila {Jennings, 2004 #2125] Spt5 are shown to disrupt the elongation repressive activity *in vitro*, a loss of repressive activity of these mutant proteins has not been demonstrated *in vivo*. In fact, neither the zebrafish point mutation nor the zygotic null mutation of *spt5* significantly de-represses *hsp70-4*, a gene that is repressed at the elongation level (Chen and Guo, unpublished observations, and this study).

The positive role of Spt5 in transcription elongation *in vivo* has been supported by its occupancy detected at many transcriptionally active chromosomal sites and its rapid recruitment to endogenous and transgenic heat shock loci upon heat shock, whereas its repressive activity has been suggested by its presence at the promoter proximal region in un-induced heat shock gene loci [34], [35]. Recently, expression profiling together with *in vivo* chromatin immunoprecipitation (ChIP) studies in the zebrafish *spt5* mutant reveal essential target genes that are occupied and regulated by Spt5, hence providing direct evidence that Spt5 indeed has dual activity in regulating RNAPII elongation *in vivo* [36].

In this study, we sought to determine how such *in vivo* dual activity of Spt5 is encoded in the protein. Through a structure-function study in zebrafish, we found that deleting either CTR1 or CTR2CT does not significantly affect Spt5's *in vivo* activity, suggesting that partially redundant functions reside in the Spt5 C-terminus. However strikingly, deleting the entire C-terminus yielded NSpt5, which had a dominant activity that impaired embryogenesis in zebrafish. Using *hsp70-4* and *HIVLTR*, two examples of RNAPII elongation -repressed genes, we found that NSpt5 acted to de-repress their transcription. *In vivo* ChIP further uncovered that NSpt5 directly associated with *hsp70-4* chromatin *in vivo* and increases the occupancy of RNAPII, P-TEFb, H3K4Me3, and surprisingly, NELF-A at the locus, indicating a direct action of NSpt5 on the elongation repressed *hsp70-4* gene *in vivo*. Together, these results reveal a dominant activity of NSpt5 to de-repress RNAPII elongation, and suggest that the C-terminus of Spt5 is critical for repressing RNAPII elongation *in vivo*.

## Results

### Redundant Activity of CTR1 and CTR2CT Domains of Spt5 in zebrafish development

**Figure 1:**
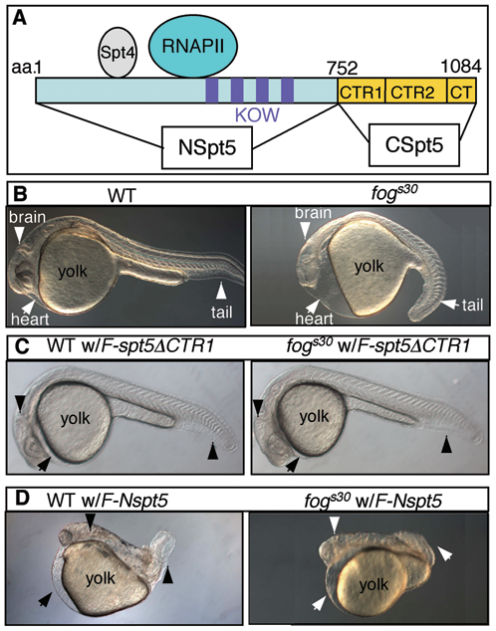
Injection of *F-Nspt5* RNA into WT dominantly impairs embryonic development in zebrafish. (A) The functional domains of Spt5 based on previous *in vitro* analysis [15], [37]. (B–E) Morphological phenotypes of WT or *fog^s30^* embryos (B), WT or *fog^s30^* embryos injected with *F-spt5* RNA (C), *F-spt5ΔCTR1* RNA (D), or *F-Nspt5* RNA (E).

To dissect the activity of Spt5 (**Figure 1A**) *in vivo*, we employed a RNA rescue assay in zebrafish (**Figure 1**). The *fog^s30^* allele (**Figure 1B**), which harbors a deletion of the entire locus of *spt5*, was used as the ground state of *spt5* activity (a zygotic null with residual maternal Spt5 activity) [26]. FLAG epitope-tagged wild type (WT) *spt5* (*F-spt5*) mRNA rescued *fog^s30^* mutant phenotypes fully, as assessed by the overall normal morphology (**Figure S1**, and **Table 1**) and the proper development of dopaminergic (DA) neurons at 30 hours post fertilization (hpf)(**Figure S2B**). *F-spt5* had no discernible effect in WT embryos, suggesting that overexpression of *spt5* alone does not interfere with its function *in vivo* (**Table 1**).

**Table 1:** The ability of deletion variants in rescuing *fog^s30^* mutant embryos and producing dominant phenotypes in WT embryos.

RNA variants injected	Ability to rescue *fogs30* morphology (%, n = a/bˆ )	Ability to produce dominant interference
*1. F-spt5*	100%, n = 7/30	0%, n>50
*2. F-spt5ΔRNAPII-BD*	0% *	0%, n>50
*3. F-spt5ΔCTR1*	100%, n = 5/38	0%, n>50
*4. F-spt5ΔCTR2CT*	100%, n = 9/35	0%, n>50
*5. F-Nspt5*	0%*	100%, n>50

ˆ a equals to the number of genotypically mutant embryos after injection, b equals to total number of embryos genotyped after injection. % means the percent mutant embryos that are rescued to WT morphology.

* The expected ∼25% mutant embryos are observed in the injected embryos, and therefore, genotyping was not carried out.

Using this *in vivo* assay, the activity of a series of *spt5* deletion variants was tested. Notably, removal of the RNAPII-binding domain [15], [37] in Spt5 (F-*spt5ΔRNAPII-BD*) abolished its ability to rescue *fog^s30^* embryos (**Table 1** and **Figure S2C**), despite the variant protein being detected readily in the embryo (data not shown). This observation suggests that the activity of Spt5 *in vivo* is mediated via its interaction with RNAPII. The expression of *F-spt5ΔRNAPII-BD* had no discernible effect in WT embryos (**Figure S2C** and **Table 1**).

Next, we turned our attention to the C-terminus of Spt5 (CSpt5), which is composed of two repeat-containing regions named CTR1 and CTR2, and a small new domain called CT (**Figure 1A**). CTR1 contains multiple hepta-peptide repeats that are phosphorylated by P-TEFb [21], and is considered as an important domain for Spt5's positive elongation activity, as the prevention of CTR1 phosphorylation impairs epidermal growth factor (EGF)-induced *c-fos* expression but not its basal transcription in Hela cells [19]. However, CTR1-deleted Spt5 (*F-spt5ΔCTR1*) fully rescued the morphological defects of *fog^s30^* embryos (**Figure 1C**) and the development of DA neurons (**Figure S2D**). Spt5 with a deletion of the other half of CSpt5 (*F-spt5ΔCTR2CT*) also fully rescued the embryonic morphology (**Table 1**) but not DA neuron development (**Figure S2E**). This is consistent with the previous published observation, in which a point mutation in CT affects DA neuron development but not embryonic morphology [25]. Taken together, CTR1 and CTR2CT appear to carry out partially redundant functions *in vivo*.

### Removal of the entire C-terminus (CSpt5) unleashes a dominant activity that resides in the N-terminus of Spt5 (NSpt5)

We next determined the functional effect of removing the entire C-terminus (CSpt5). Spt5 with both CTR1 and CTR2CT deleted (NSpt5) not only failed to rescue *fog^s30^* mutant phenotypes, including the body shape, brain morphology, eyes, heart, and blood circulation, but also exacerbated them. Moreover, NSpt5 impaired dominantly the development of WT embryos (**Figure 1D**, and **Table 1**). WT embryos injected with *F-Nspt5* RNA (*F-Nspt5*-expressing embryos) exhibited a similar phenotype to NSpt5-expressing *fog^s30^* embryos, with severe deformity including small size, deformed brain and eyes, heart edema, lack of blood circulation, and dorsally curved body axis (**Figure 1D**), with the phenotypes worsening when more *F-Nspt5* RNA was injected. No significant increase in cell death was detected in *NSpt5*-expressing embryos (data not shown). These results suggest that Nspt5 has no rescuing activity, but exhibits a dominant negative effect on the endogenous Spt5 (both the normal levels of Spt5 in WT and residual maternal Spt5 in the *fog^s30^* mutant).

### NSpt5 de-represses *hsp70* expression in the absence of heat shock but does not affect the induction of *hsp70-4* upon heat shock

To understand NSpt5's dominant activity, we carried out gene expression profiling on WT and NSpt5-expressing embryos. We identified a number of genes whose transcript levels were significantly increased in NSpt5-expressing embryos (H. Chen, unpublished data). One of the most prominent is *hsp70-4*: its transcript level was increased ∼10 fold in NSpt5-expressing embryos. Since *hsp70* is a model gene whose transcription elongation is regulated by Spt5 [2], we focused our analysis on *hsp70-4* in this study. Multiple members of the *hsp70* gene family were identified in zebrafish, and *hsp70-4* was shown to be heat inducible [38]. A GFP reporter line driven by *hsp70-4* regulatory elements mimics the endogenous *hsp70-4* expression [39], and moreover, the induction of GFP upon heat shock in this line was attenuated in *fog^s30^* embryos [26], suggesting that its expression is under Spt5 regulation.

**Figure 2:**
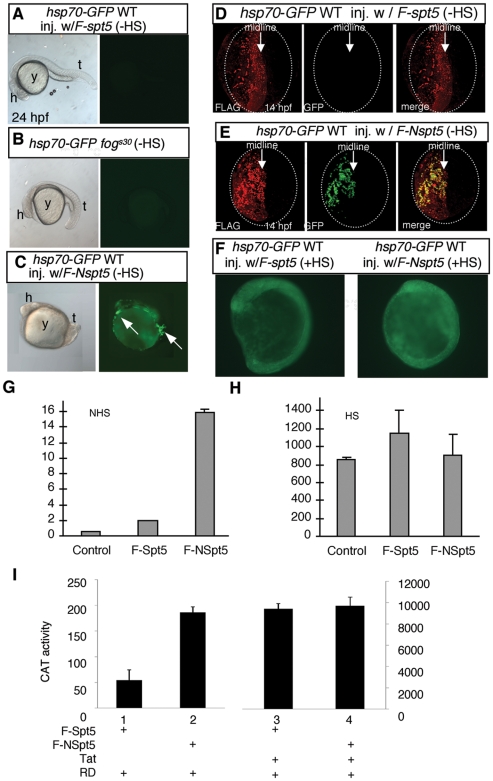
NSpt5 de-represses *hsp70-4* expression in the absence of heat shock. (A–C) Embryonic morphology or GFP fluorescence of *hsp70-GFP* transgenic embryos. *hsp70-GFP* transgenic WT injected with *F-spt5* RNA (A), *hsp70-GFP* transgenic *fog^s30^* mutant (B), and *hsp70-GFP* transgenic WT injected with *F-Nspt5* RNA (C). (D–E) Confocal images of FLAG- and GFP- double immuno-labeled embryos, injected with *F-spt5* RNA (D), or with *F-Nspt5* RNA (E). (F) GFP fluorescence in *hsp70-GFP* transgenic embryos injected with *F-spt5* RNA (left) or *F-Nspt5* RNA (right) and subjected to heat shock for one hour. (G–H) Quantitative RT-PCR analysis shows de-repression of *hsp70-4* expression in 6 hpf *Nspt5*-expressing embryos (G), and no significant difference of *hsp70-4* expression between *F-Nspt5*-expressing, *F-Spt5*-expressing, and control embryos upon heat shock (H). (I) F-NSpt5 increases transcription from the *HIVLTR*. CAT activity of Hela cells that express RD and F-Spt5 (lanes 1 and 3), or F-NSpt5 (lanes 2 and 4), in the absence (lanes 1 and 2) or presence of Tat (lanes 3 and 4). Results are presented in arbitrary units. Error bars represent S.E.M. from three independent experiments.

We first examined the impact of NSpt5 on *hsp70*-GFP expression. In the absence of heat shock, no GFP signal was detected in WT (**Figures 2A and D**) or in *fog^s30^* embryos (**Figure 2B**). Although our previous studies did detect ∼2 fold increase of basal *hsp70-4* transcripts in the *fog^sk8^* mutant [36], such modest increase might not cause sufficient accumulation of GFP protein to be above the detection sensitivity of the fluorescent microscope. However, in WT embryos expressing *F-Nspt5*, a strong GFP signal was detected (**Figures 2C and E**), often in patches of embryonic cells. The patchiness of the signal could possibly be due to transient and mosaic expression of the injected *F-Nspt5* RNA, or alternatively, due to unknown spatial and temporal constraints on *hsp70-4* expression in developing embryos. Double labeling with FLAG and GFP antibodies revealed that the GFP signal was detected in cells that co-expressed the FLAG epitope, indicating a cell-autonomous effect of F-NSpt5 (**Figure 2E**). Upon heat shock for 1 hour, *hsp70*-GFP was induced equally well in *F-spt5*- or *F-Nspt5*-expressing embryos (**Figure 2F**).

The effect of NSpt5 on endogenous *hsp70-4* expression was further confirmed by quantitative RT-PCR and *in situ* hybridization. About 14 fold increase of *hsp70-4* transcripts were detected in *Nspt5*-expressing embryos (**Figure 2G**). *In situ* hybridization further revealed the increase and the spatial distribution of *hsp70-4* transcripts in *Nspt5*-expressing embryos (**Figure S3**). With heat shock, *hsp70-4* expression was dramatically increased, and such heat shock-induced *hsp70-4* expression was unaffected by *Nspt5* mis-expression (**Figure 2H**). Together, these results suggest that NSpt5 can interfere with the elongation repressive activity of Spt5 (revealed by the increase of basal *hsp70-4* expression), but not the elongation stimulatory activity of Spt5 (revealed by the un-alteration of heat shock-induced *hsp70-4* expression).

### NSpt5 de-represses *HIVLTR* transcription under basal conditions but does not affect Tat trans-activation in human cells

To determine whether NSpt5's effect is more general than one gene (*hsp70-4*) and one species (zebrafish), we examined *HIVLTR*, which harbors a promoter-proximal stalled RNAPII [5] and Spt5 [40] in human Hela cells. In the absence of the viral transactivator Tat, NELF and DSIF cooperatively promote the arrest of RNAPII on the *HIVLTR*, resulting in the accumulation of short transcripts [41]. However, when Tat is recruited to the transactivation response (TAR) RNA, Spt5 helps to increase rates of productive elongation [41]–[43]. Since Spt5 from zebrafish is homologous in structure and function to human Spt5 protein [25], F-Spt5 or F-NSpt5 were co-expressed with RD (a component of NELF) in HeLa cells in the absence or presence of Tat (**Figure 2I**), and the activity of chloramphenicol acetyltransferase (CAT) fused to *HIVLTR* was examined. In the absence of Tat, the expression of F-NSpt5 resulted in almost a four fold increased expression from the *HIVLTR* (**Figure 2I** **,** bar 2) as compared to that of F-Spt5 control (**Figure 2I** **,**, bar 1). In the presence of Tat, there was no significant difference between F-Spt5- or F-NSpt5- mediated CAT activity (**Figure 2I** **,** bars 3 and 4). These results suggest that NSpt5 again interferes with Spt5's repressive but not its stimulatory activity in *HIVLTR* transcription, similar to its action on *hsp70-4*.

### NSpt5 impairs the repressive but not the stimulatory activity of DSIF in a dominant manner *in vitro*

**Figure 3:**
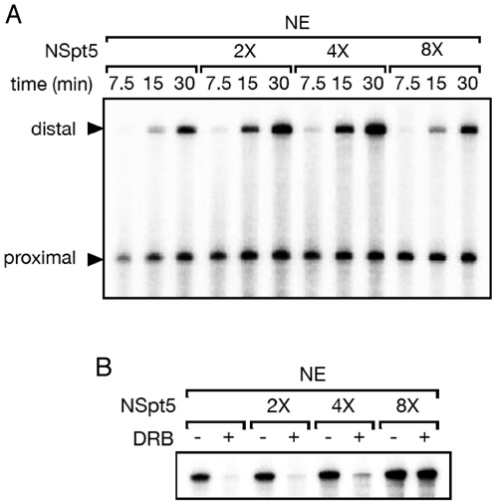
NSpt5 interferes with the repressive but not the stimulatory activity of endogenous DSIF in a dominant manner *in vitro*. (A) The elongation stimulation activity of DSIF was assayed using pSLG402 as a template, which generates short (promoter-proximal) and long (promoter-distal, dependent on the elongation stimulatory activity of DSIF) RNase T1-resistant products under the control of the adenovirus major-late promoter. Transcription initiation/elongation was allowed to proceed for the indicated times. Time-dependent increase of distal transcripts is observed in the control (first three lanes), while NSpt5 slightly enhanced transcription at 2X or 4X concentration but not at 8X concentration. (B) pTF3-6C2AT, which generates a 380-nt RNase T1-resistant product under the control of the adenovirus E4 promoter, was used as a template, and transcription was allowed to proceed for 10 minutes. This product is sensitive to the elongation repressive activity of DSIF (in the presence of the P-TEFb inhibitor DRB)(first two lanes). NSpt5 inhibits the repression activity of endogenous DSIF at 8X concentration.

To further test the idea that NSpt5 interferes with Spt5's repressive but not its stimulatory activity, we employed an *in vitro* system, which measures Spt5's activating activity in the presence of constitutively active P-TEFb and its repressive activity upon P-TEFb inactivation via the kinase inhibitor DRB [12]. The elongation stimulation activity of DSIF was assayed using pSLG402 as a template, which generates short (promoter-proximal) and long (promoter-distal) RNase T1-resistant products under the control of the adenovirus major-late promoter. The long (promoter-distal) transcripts are dependent on the elongation stimulatory activity of DSIF. Nuclear extracts (NE) containing the constitutively active P-TEFb and WT DSIF led to a time-dependent increase of the long (promoter-distal) RNase T1-resistant products, reflecting the elongation stimulatory activity of Spt5 (**Figure 3A** **, first three lanes**). Increasing amounts of NSpt5 were added to normal HeLa nuclear extract and were found to modestly enhance elongation efficiency (**Figure 3A**). This may be due to its dominant-negative effect on the repressive activity of endogenous DSIF, but this could also be explained by its residual elongation activation potential. To clarify this point, we used a different transcription system, in which synthesis of a 380-nt RNase T1-resistant product was examined in the presence or absence of the transcriptional inhibitor DRB (**Figure 3B**). DRB blocks P-TEFb kinase activity, and therefore the repression activity is clearly seen in the presence of DRB (Figure 3B, first two lanes), whereas its activation activity is not clearly seen because of relatively short transcript length. Consequently, while NSpt5 had only a modest effect in the absence of DRB, NSpt5 strongly interfered with the transcription inhibition by DRB (**Figure 3B**), suggesting that NSpt5 indeed acts in a dominant-negative manner, capable of inhibiting the repressive activity of endogenous DSIF. These findings provide biochemical evidence that NSpt5's dominant activity *in vivo* is due to its ability to interfere with endogenous Spt5's repressive but not its stimulatory activity.

### NSpt5 increases RNAPII occupancy on the *HIVLTR* and *hsp70-4* chromatin in human cells and zebrafish

**Figure 4:**
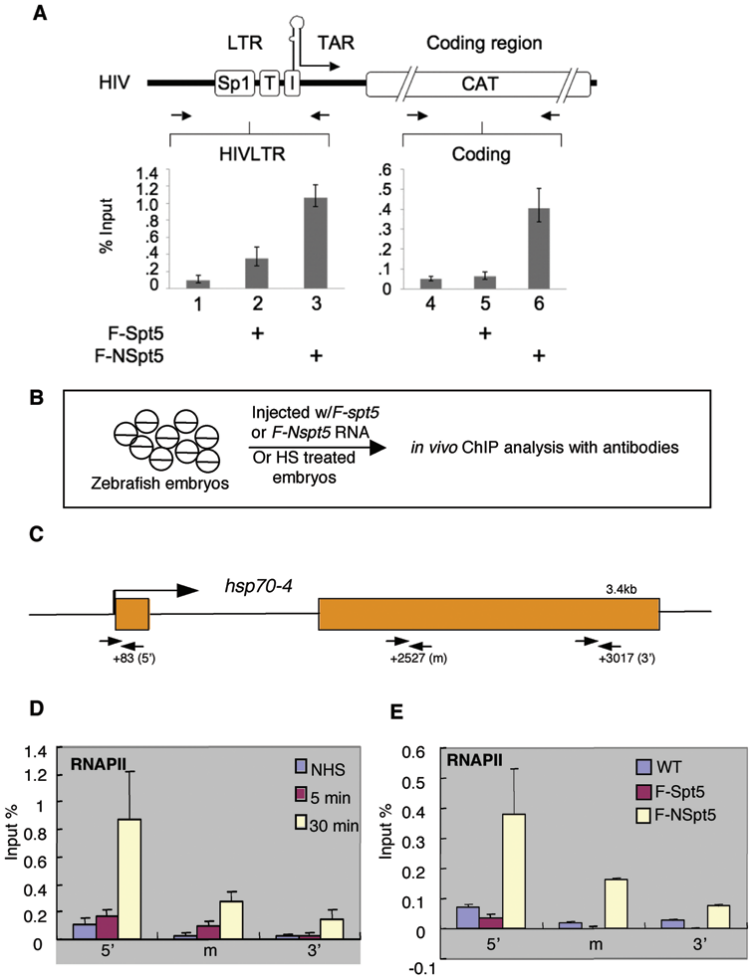
RNAPII elongates on the *HIVLTR* and *hsp70-4* chromatin in the presence of F-NSpt5. (A) ChIP and qRT-PCR were performed with RNAPII antibodies and indicated primers (arrows) in Hela cells. Data are presented as percent of input material immunoprecipitated with specific antibodies over those with the IgG control. Error bars represent S.E.M. of triplicate measurements from three independent experiments. (B) A scheme of the *in vivo* ChIP analysis in zebrafish. (C) The gene structure of *hsp70-4*, highlighting the location of the *hsp70-4* primer sets that are used during real-time PCR amplification of the immuoprecipitated material. The long arrow indicates the start of transcription. (D) The relative enrichment of RNAPII on *hsp70-4* with RNAPII antibody over the IgG control under heat shock condition. (E) The relative enrichment of RNAPII on *hsp70-4* in NSpt5 expressing embryos. Error bars represent S.E.M. of duplicate measurements from two independent experiments.

To further examine NSpt5's mechanism of action in intact cells, we carried out chromatin immunoprecipitation (ChIP) studies, first in Hela cells, to determine whether NSpt5 impacted the occupancy of RNAPII on *HIVLTR* chromatin in the absence of Tat (**Figure 4A**). To investigate promoter proximal and distal transcription, we used anti-RNAPII antibodies and PCR primers specific for the *HIVLTR* and CAT coding sequences (**Figure 4A**, top panel). F-NSpt5, but not F-Spt5, significantly increased the presence of RNAPII on the *HIVLTR* and CAT coding regions (**Figure 4A**, compare bars 2 and 5 to bars 3 and 6). Taken together, these results suggest that NSpt5 significantly enhances RNAPII occupancy on the *HIVLTR* in the absence of the Tat trans-activator.

We next carried out ChIP studies to examine the occupancy of RNAPII on the *hsp70-4* chromatin in zebrafish embryos (**Figure 4B**). The *hsp70-4* locus in zebrafish is ∼3.4 kb in length, and contains two exons and one intron (**Figure 4C**). We first determined the patterns of RNAPII on *hsp70-4* in WT embryos with or without heat shock. In WT embryos without heat shock, RNAPII (**Figure 4D**) exhibited a higher occupancy at the 5′ than the 3′ end, which is similar to the occupancy pattern of RNAPII on the *hsp70* locus in Drosophila, indicating that there is an elongation block at the promoter proximal region. Upon heat stimulus of various lengths of time, occupancies of RNAPII (**Figure 4D**) are significantly increased at the 5′end and moderately increased at the downstream region, which is consistent with the observed persistence of elongation pause under heat shock condition in Drosophila [44]. In *Nspt5*-expressing embryos, the increased occupancy pattern of RNAPII was similar to the pattern observed under heat shock, although the occupancy level is lower than that with heat shock (**Figure 4E**). In *spt5*-expressing embryos, RNAPII occupancy was not increased (**Figure 4E**). Together, these results suggest that NSpt5 de-represses the expression of *HIVLTR* and *hsp70-4* in the non-induced conditions, through increasing RNAPII occupancy at these loci.

### NSpt5 directly interacts with the *hsp70-4* chromatin *in vivo*

**Figure 5:**
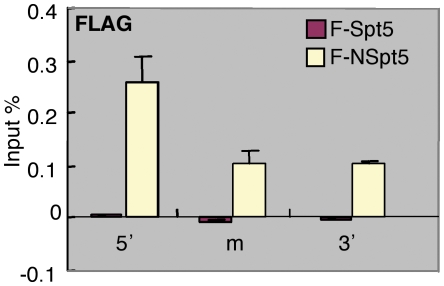
NSpt5 directly interacts with the *hsp70-4* chromatin *in vivo*. Charts show the percent of input material immunoprecipitated in different regions of *hsp70-4* chromatin. The relative enrichment of ChIP and qRT-PCR values obtained with Flag antibody over the IgG control. Error bars represent S.E.M. of duplicate measurements from two independent experiments.

To determine whether NSpt5 directly or indirectly causes the enhanced RNAPII occupancy on the *hsp70-4* chromatin *in vivo*, we examined the chromatin occupancy of NSpt5, in comparison with Spt5. ChIP was carried out using the anti-FLAG antibody in *F-spt5*- or *FNspt5*- expressing embryos. F-NSpt5 was found to associate with the *hsp70-4* chromatin (**Figure 5**). However, F-Spt5 did not exhibit any detectable association with the *hsp70-4* chromatin (**Figure 5**). This observation suggests that NSpt5 directly interacts with the *hsp70-4* chromatin to cause the increased RNAPII occupancy at the locus. The fact that the exogenously provided Spt5 has no association with the *hsp70-4* chromatin suggests that NSpt5 has better access to the *hsp70-4* chromatin than the full length Spt5.

### NSpt5 increases the occupancy of Cdk9, histone marks for active transcription, and NELF-A at the *hsp70-4* promoter region *in vivo*

**Figure 6:**
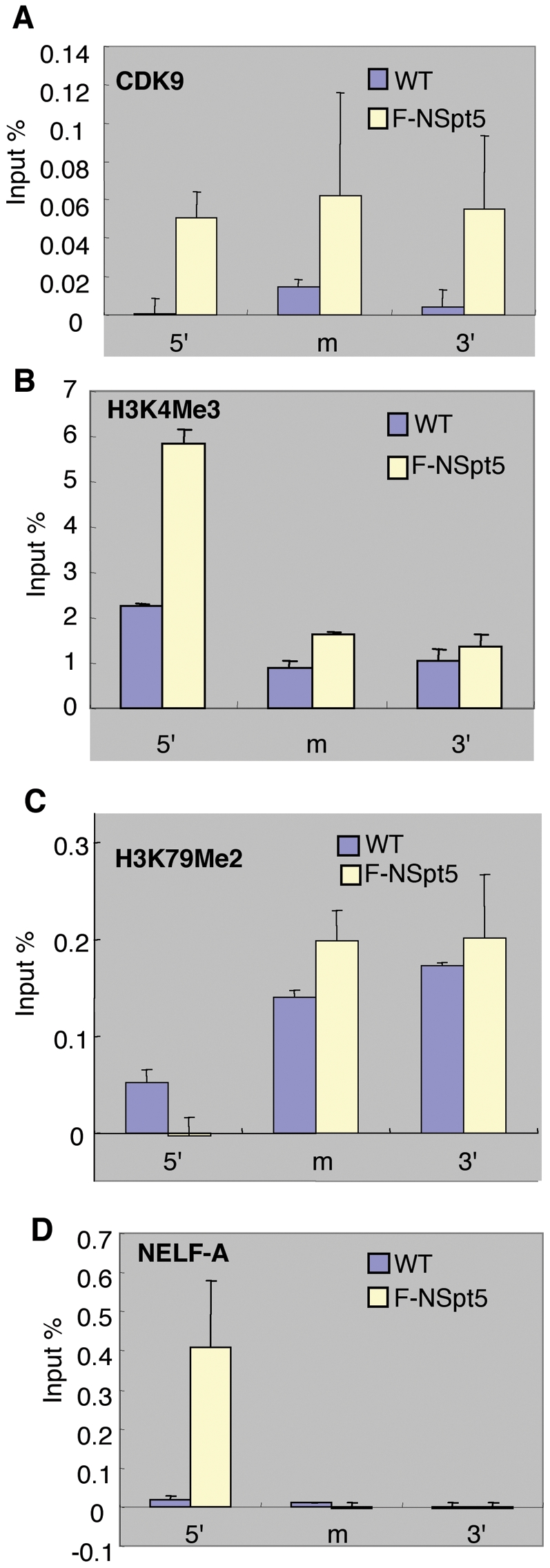
ChIP at the *hsp70-4* chromatin in *Nspt5* expressing zebrafish embryos. (A to D) Charts show the percent of input material immunoprecipitated in different regions of *hsp70-4* chromatin. The relative enrichment of ChIP and qRT-PCR values obtained with CDK9 antibody over the IgG control (A), H3K4Me3 antibody over the IgG control (B), or H3K79Me2 antibody over the IgG control (C), NELF-A antibody over the rabbit serum control (D). Error bars represent S.E.M. of duplicate measurements from two independent experiments.

We next determined the *in vivo* chromatin occupancy of other proteins involved in regulating transcription elongation at the *hsp70-4* locus *in vivo*. Cdk9 is the kinase subunit of P-TEFb. In the presence of NSpt5, the occupancy of Cdk9 is significantly increased at the 5′ end, middle, and 3′ end (**Figure 6A**). Inhibition of Cdk9 activity, either through a specific morpholino antisense oligonucleotide or using the kinase inhibitor flavoperidol (FP), significantly suppressed NSpt5-induced dorsalization phenotype and the de-repression of *hsp70-4* (**Figure S4 and S5**). Several lines of evidence suggest that such a suppression of NSpt5 effects is not due to a general developmental delay but rather, is likely due to a direct requirement of CDK9 in the manifestation of NSpt5's dominant negative effect: First, we checked later stages of Nspt5-expressing and Cdk9-impaired embryos, and still did not see a dorsalization phenotype, suggesting that it is not due to a simple developmental delay. Second, at the molecular level, NSpt5-mediated, increased expression of *hsp70-4*, which is a marker gene directly regulated by Spt5, is significantly impaired by Cdk9 knockdown. Third, Cdk9, being a subunit of P-TEFb, is known to phosphorylate RNAPII CTD, Spt5 C-terminal domains, and NELF, which together form protein complexes, suggesting a possible direct requirement of RNAPII or NELF phosphorylation for the manisfestation of NSpt5's effect. Taken together, we suggest that Cdk9 activity and hence the phosphorylation of RNAPII or NELF is likely to be directly required for NSpt5's dominant negative effect *in vivo*. Consistent with the increased occupancy of Cdk9, we also observed increased occupancy of H3k4Me3 (**Figure 6B**), and a slight increase in occupancy of H3K79Me2 (**Figure 6C**), both of which represent histone marks for active transcription [45].

DSIF collaborates with RNAPII and NELF complex to stall transcription elongation *in vitro* [13]. Since upon heat shock induction, DSIF and RNAPII but not NELF are strongly recruited to chromosomal puffs harboring the *hsp70* genes [46], NELF has been viewed to have only a repressive role on RNAPII elongation. To determine the occupancy of NELF on *hsp70-4* chromatin in the presence of NSpt5, we carried out CHIP with the antibody recognizing NELF-A, a subunit of NELF (antibodies against other NELF subunits do not work in zebrafish). In control embryos, NELF-A was detected mainly at the 5′ end of *hsp70-4* chromatin, consistent with the role of NELF in the promoter proximal pause of *hsp70* (**Figure 6D**). However strikingly, in NSpt5-expressing embryos, NELF-A was dramatically and exclusively increased at the 5′end of *hsp70-4* (**Figure 6D**). This surprising result suggests that NSpt5 promotes the recruitment of NELF-A. Thus, NELF appears to behave very differently in transcriptional increases mediated by NSpt5 (this study) versus that mediated by heat shock [46], which is consistent with the significant difference of the expression level of *hsp70-4* under these two conditions (see **Figure 2G and H**).

## Discussion

Accumulating biochemical evidence suggests that the evolutionarily conserved protein Spt5 plays a critical role in regulating RNAPII elongation, by acting as a repressor or an activator under different *in vitro* assay conditions [12]. The conversion of Spt5 from a repressor to an activator involves P-TEFb, which phsosphorylate RNAPII CTD [20], [37] as well as the CTR1 domain of Spt5 [15]–[19], leading to the hypothesis that CTR1 acts as a mini-CTD for assembling active elongation complexes [19]. Despite these findings, regulation of RNAPII elongation by Spt5 *in vivo* is not well understood.

In this study, we demonstrate that CSpt5 composed of partially redundant CTR1 and CTR2CT domains is a repressive module, by showing that NSpt5 lacking this module exerts a dominant negative effect on the repressive but not the stimulatory activity of the endogenous Spt5, while has no stimulatory activity on its own. First, NSpt5 has no rescuing activity but instead dominantly impair the development of WT embryos, suggesting that NSpt5 is likely to be a dominant negative form. Second, the increase of *hsp70* expression in NSpt5-expressing WT is much lower compared to heat shock induced *hsp70* expression, suggesting that Nspt5 “de-represses” *hsp70* due to dominant negative interference with the endogenous Spt5's repressive activity, while has no elongation stimulatory activity on its own. This is consistent with a previous study, which shows that phosphorylated Spt5 C-terminus is critical for elongation stimulatory activity [19]. Third, biochemical analysis shows that NSpt5 can dominantly interfere with Spt5's repressive but not stimulatory activity. The fact that Nspt5 does not affect heat-induced *hsp70* expression or TAT transactivation at HIVLTR provides *in vivo* evidence that NSpt5 does not affect the stimulatory activity of endogenous Spt5. Finally, consistent with the dominant nature, *in vivo* ChIP analysis shows that NSpt5 has a preferential access to chromatins than WT Spt5. Because mis-expression of NSpt5 significantly impairs embryogenesis, this observation suggests that other important developmental genes are also de-repressed by NSpt5 possibly via similar mechanisms uncovered for *hsp70-4* and *HIVLTR*.

**Figure 7:**
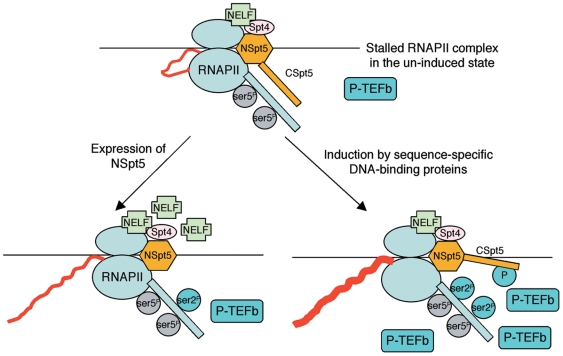
A model depicts the role of Spt5 in regulating RNAPII elongation *in vivo*. (Top) Stalled RNAPII complex on a gene that is subjected to elongation regulation, in an un-induced state. (Bottom left) RNAPII complex with the incorporation of NSpt5. (Bottom right) RNAPII complex in an induced state.

Taken previous studies and our new findings into consideration, we propose the following model to explain the role of Spt5 in regulating RNAPII elongation *in vivo* (**Figure 7**). 1) In the un-induced state, Serine-5 phosphorylated RNAPII is stalled after the synthesis of a short stretch of RNA, via an Spt5-dependent mechanism. It is conceivable that low amount of P-TEFb may be present near the locus, either because of its constitutive presence in the nucleoplasm, or due to “spill-over” from neighboring, actively transcribing loci *in vivo*. We propose that CSpt5 may play an important role to prevent the RNAPII CTD from being phosphorylated by P-TEFb, thereby repressing RNAPII elongation and at the same time allowing critical processes such as mRNA capping to occur. 2) The incorporation of mis-expressed NSpt5 into the stalled RNAPII complex removes CSpt5's repressive activity, thereby allowing Serine-2 phosphorylation by P-TEFb on the RNAPII CTD, hence transcription elongation. Alternatively, since NSpt5 has a stretch of acidic residues from aa. 3 to aa. 105, it may stimulate RNAPII transcription, through recruiting P-TEFb either directly or indirectly to the RNAPII complex. However, it is important to note that the increased transcription by NSpt5 appears much less than that in the induced state. 3) Upon induction mediated by sequence-specific DNA binding proteins, a much larger amount of P-TEFb is recruited to the locus, which can phosphorylate CSpt5 (and NELF) to remove the repressive effect, which in turn allows the phosphorylation of RNAPII CTD, thereby allowing assembly of additional accessory factors for productive elongation. Taken together, our findings reveal a previously unknown role of CSpt5 in repressing RNAPII elongation *in vivo*. It remains possible that the phosphorylated CSpt5 may have a role in positively regulating transcription elongation through recruiting active elongation complexes. Future experiments are needed to test this and further verify the validity of this model.

We have made an interesting observation that enhanced NELF-A presence coincides with increased transcription caused by NSpt5. Since NELF has been considered to inhibit transcription elongation based on biochemical studies *in vitro* [13], [47]–[49] and the colocalization of NELF with an elongation incompetent form of RNAPII on polytene chromosomes [46], it is an unexpected finding. However, NELF is recently found to be critical for enhancing gene expression by blocking promoter proximal nucleosome assembly [50], and is also found to associate with many highly expressed genes [51]. Our finding of the increased enrichment of NELF-A at the promoter region of *hsp70-4* chromatin in *Nspt5*-expressing embryos thus supports such positive role of NELF in transcription elongation.

## Materials and Methods

### Fish stocks and maintenance

Fish breeding and maintenance were performed as previously described [52]. Embryos were raised at 28.5°C and staged according to Kimmel *et al.* [53]. Fish heterozygous for the *foggy^s30^* mutation or *hsp70-GFP* transgene were bred to obtain homozygous embryos for analysis. *fog^s30^* mutant embryos were identified by their morphological defect [26] and genotyped for the lack of genomic DNA encoding *spt5*. Transgenic *hsp70-GFP* embryos were identified based on the green fluorescent lenses that are apparent at 48 hpf and/or based on their ability to up-regulate GFP upon heat shock [39].

### mRNA synthesis, morpholino, and injections

mRNAs were synthesized from the following plasmids: *pCS2-F-spt5*, *pCS2-F-spt5ΔRNAPII-BD* (Δaa.313-513), *pCS2-F-spt5ΔCTR1* (Δaa.752-815), *pCS2-F-spt5ΔCTR2CT* (aa.1-815), *pCS2-F-Nspt5* (aa.1-751) and injected at 200–500 ng/µl with 2–3 nl into the yolk of one- to eight-cell-stage embryos as previously described [54].

*CDK9* Morpholino (MO) antisense oligonucleotide (Gene Tools, Corvallis OR) was designed to complement the exon 2/intron 2 junction. The MO sequence was: ACATCAAATACTCACCCAAAGGTGC. 1–2 nl of the morpholino oligonucleotide was injected at a concentration of 1.25 mM.

### Immunohistochemistry

Immunohistochemistry was performed as previously described [54]. The following primary antibodies were used: rabbit polyclonal anti-GFP (Abcam), mouse anti-Flag M2 antibody (Sigma). The following secondary antibodies were used: anti-rabbit IgG Alexa Fluor 488 and anti-mouse Alexa Fluor 568 (Molecular Probes). Differential interference contrast microscopy was performed on a Zeiss Axiophot 2 microscope and fluorescence microscopy was performed using a Leica TCS SP2 confocal microscope.

### Chromatin immunoprecipitation (ChIP) in zebrafish embryos

De-chorionated embryos (about 150 at 9 hpf) were collected and cross-linked with 1% formaldehyde for 20 min at room temperature and quenched by addition of glycine to 0.125 M final concentration for 5 min. After being washed twice with PBS, the embryos were snap-frozen and stored at −70°C. The embryos were homogenized and resuspended in 300 µl lysis buffer (10 mM Tris-HCl pH 8.0, 200 mM NaCl, 10 mM EDTA, 0.5% NP-40, 0.1% SDS and protease inhibitor cocktail from Roche). The suspension was then sonicated on ice to generate approximately 500 base-pair (bp) fragments. The lysates were centrifuged, pre-cleared with protein-A or protein-G agarose beads (Sigma), and then divided into 0.15 ml aliquots per immunoprecipitation (5% of the lysate was kept as ‘input’ before the addition of the antibody). Antibodies for immunoprecipitation were as follows: mouse anti-RNA Pol II (8WG16, Covance Research Products); mouse anti-Flag M2 antibody (Sigma), and rabbit anti-Spt5 polyclonal antibody raised against the zebrafish C-terminal region (CTR1CTR2CT). After addition of the antibody, lysates were incubated at 4°C overnight, and then incubated with protein-A or protein-G agarose beads (30 µl resin) for 1 hr at 4°C. The beads were washed once with DNA wash buffer (10 mM Tris-HCl pH 8.0, 200 mM NaCl, 10 mM EDTA, 0.5% NP-40), once with DNA wash buffer containing 100 µg/ml salmon sperm DNA (Invitrogen), once with 5×RIPA buffer (10 mM Tris-HCl pH 8.0, 500 mM NaCl, 1 mM EDTA, 0.5 mM EGTA, 0.1% SDS, 1% Triton, 0.1% Sodium deoxycholate), and once with LiCl buffer (10 mM Tris-HCl pH 8.0, 250 mM LiCl, 1 mM EDTA, 0.5 mM EGTA, 1% Triton, 1% sodium deoxycholate). Finally, the beads were washed once with DNA wash buffer and pelleted, and chromatin was eluted from the beads by adding 160 µl elution buffer (25 mM Tris-HCl pH 7.5, 10 mM EDTA and 0.5% SDS) and incubated at 65°C for 20 min, then eluted with 100 µl elution buffer again. The inputs were also added with the elution buffer to 260 µl. After addition of equal volume digestion buffer (50 mM Tris-HCl pH 8.0, 1 mM EDTA, 100 mM NaCl and 0.5% SDS, 0.09 mg/ml proteinase K and 0.1 mg/ml RNase A), all samples were incubated at 42°C for 1 hr and 65°C overnight to reverse cross-linking. Chromatin DNA was purified by phenol extraction followed by alcohol precipitation, and used for real-time PCR.

The following primers were used:

5′-F (−11), 5′-CCAGCATAGACTTCGCGATAGAAC-3′;

5′-R (+83), 5′-AACAAGCCATCAATACGCCTGAC-3′;

m-F (+2366), 5′-TCATCAAGCGCAACACAACCATCC-3′;

m-R (+2527), 5′-AGGTGGAATTCCCGTCAGGTCAAA-3′;

3′-F (+2801), 5′-CCTGGAGTCTTACGCCTTCAACATG-3′;

3′-R (+3017), 5′-TCCCTGGTAGAGTTTGGAGATGACTG-3′;

Numbers in parentheses represent positions relative to the transcription initiation site of *hsp70-4*.

### Analysis of *hsp70-4* mRNA levels

The control or injected embryos were collected at 6 hpf and total RNA was extracted using Trizol reagent (Invitrogen) and treated with Turbo DNA-*free* DNase (Ambion). First-strand cDNA was reverse transcribed using oligo-dT primers and Superscript reverse transcriptase (Invitrogen). Real-time PCR amplifications of *hsp70-4* and *β-actin* were carried out with SYBR Green PCR Master Mix (Applied Biosystems). Primers used were Actin-forward (F), 5′-TGAGCGCAAATACTCCGTCTGGAT-3′, Actin-reverse (R), 5′-GTTCGAGAGTTTAGGTTGGTCGTTCG-3′ and *hsp70-4* 3′end primers that were used in the ChIP experiment.

### Transfections and CAT assays

HeLa cells were co-transfected with of pEF-RD (0.5 µg) and/or pCMV-SPT5 (wild-type or mutant version, 0.5 µg) and pHIV-CAT (0.1 µg), in the absence or presence of pTat (0.1 µg) using Fugene 6 according to the manufacturer's instruction (Roche). 48 hours after the transfection, the activity of chloramphenicol acetyltransferase (CAT) was measured in the cell lysate by using a Lumitech ReportaLight Bioassay kit (Cambrex Bioscience).

### ChIP in Hela cells

ChIP was carried out essentially as described previously [55]. Primers used for LTR were described previously [56] and primers for CAT are: forward-5′-atcccaatggcatcgtaaag-3′; reverse-5′-tcgtcgtggtattcactcca-3′. Standard curves for each primer pair were obtained first to determine their amplification efficiencies. Products were quantified using Brilliant SYBR Green qPCR according to manufacturer's directions (Stratagene). Relative enrichment was calculated and normalized to the input.

Additional methods can be found online as “Supplementary Methods S1”

## Supporting Information

Figure S1 (0.76 MB PDF)

Figure S2 (9.36 MB DOC)

Figure S3 (2.74 MB DOC)

Figure S4 (0.04 MB DOC)

Figure S5 (3.17 MB DOC)

Methods S1 (0.02 MB DOC)
