# Supplementary material for: Repression of RNA Polymerase II Elongation In Vivo Is Critically Dependent on the C-Terminus of Spt5
Source: PLoS One. 2009 Sep 9;4(9):e6918. doi: 10.1371/journal.pone.0006918 (PMC2735033; doi:10.1371/journal.pone.0006918)
Supplement: Figure S1 — (0.76 MB PDF) [file pone.0006918.s001.pdf]

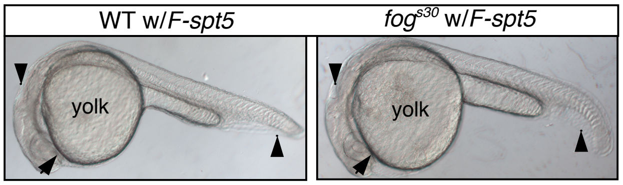

Figure S1. Morphological phenotypes of WT or *fog*^{s30} embryos injected with *F-spt5* RNA. Injection of *F-spt5* RNA fully rescues *fog*^{s30} embryo (right), but does not interfere with embryonic development in zebrafish WT embryo (left).
