# Supplementary material for: Repression of RNA Polymerase II Elongation In Vivo Is Critically Dependent on the C-Terminus of Spt5
Source: PLoS One. 2009 Sep 9;4(9):e6918. doi: 10.1371/journal.pone.0006918 (PMC2735033; doi:10.1371/journal.pone.0006918)
Supplement: Figure S2 — (9.36 MB DOC) [file pone.0006918.s002.doc]

**Figure S2. The effect of Spt5 deletion variants on DA neuron development.** All images are lateral view of embryos in situ hybridized with the *tyrosine hydroxylase* (*th*) probe. Anterior is to the left, and dorsal is up. Abbreviations: DA, dopaminergic neurons; fb, forebrain; hb, hindbrain; LC, locus coeruleus; mb, midbrain; NA, noradrenergic neurons.
