# Supplementary material for: Repression of RNA Polymerase II Elongation In Vivo Is Critically Dependent on the C-Terminus of Spt5
Source: PLoS One. 2009 Sep 9;4(9):e6918. doi: 10.1371/journal.pone.0006918 (PMC2735033; doi:10.1371/journal.pone.0006918)
Supplement: Figure S3 — (2.74 MB DOC) [file pone.0006918.s003.doc]

WT

WT inj. w/*F-spt5*

WT inj.w/*F-Nspt5*

*hsp70*

6 hpf

*hsp70*

6 hpf

*hsp70*

6 hpf

*hsp70*

9 hpf

*hsp70*

9 hpf

*hsp70*

9 hpf

*hsp70*

14 hpf

*hsp70*

*hsp70*

y

y

y

y

y

14 hpf

14 hpf

**Figure S3. *In situ* hybridization showing *hsp70-4* expression.** Increased *hsp70-4* expression in WT injected with *F-Nspt5* RNA (right, arrows), as compared to WT (left), or WT injected with *F-spt5* RNA (middle), at three different developmental stages. Abbreviation: y, yolk.
