# Supplementary material for: Repression of RNA Polymerase II Elongation In Vivo Is Critically Dependent on the C-Terminus of Spt5
Source: PLoS One. 2009 Sep 9;4(9):e6918. doi: 10.1371/journal.pone.0006918 (PMC2735033; doi:10.1371/journal.pone.0006918)
Supplement: Figure S4 — (0.04 MB DOC) [file pone.0006918.s004.doc]

**Figure S4. The efficacy of *CDK9* splicing morpholino in impairing the splicing of *CDK9* transcript.** (**A**) The exon/intron structure of *CDK9* gene, with the position of MO targeting region and the primers used to assess splicing indicated. (**B**) Quantitative RT-PCR using F1 and R1 primers shows increased retention of intron 2.
