# Supplementary material for: Repression of RNA Polymerase II Elongation In Vivo Is Critically Dependent on the C-Terminus of Spt5
Source: PLoS One. 2009 Sep 9;4(9):e6918. doi: 10.1371/journal.pone.0006918 (PMC2735033; doi:10.1371/journal.pone.0006918)
Supplement: Figure S5 — (3.17 MB DOC) [file pone.0006918.s005.doc]

**Figure S5.** **P-TEFb is required for NSpt5’s *in vivo* effects.** (**A-D**) The morphological phenotypes of CDK9 morpholino-injected (B), NSpt5-expressing (C), or CDK9 morpholino-injected and NSpt5-expressing (D) embryos. (**E-F**) flavopiridol (FP) treated (E) embryos as compared to FP treated and NSpt5-expressing embryos (F). (**G-I**) The morphological phenotypes of CDK9 morpholino-injected (G), NSpt5-expressing (H), or CDK9 morpholino-injected and NSpt5-expressing (I) embryos at later developmental stages. These studies show that impairment of CDK9 activity partially suppressed the dorsalization phenotype of NSpt5-expressing embryos. (**J-K**) Quantitative RT-PCR analyses show that both *CDK9* MO and FP treatment decreases *hsp70* transcripts induced by NSpt5. Abbreviations, h, head; t, tail; y, yolk.
