# Supplementary material for: Repression of RNA Polymerase II Elongation In Vivo Is Critically Dependent on the C-Terminus of Spt5
Source: PLoS One. 2009 Sep 9;4(9):e6918. doi: 10.1371/journal.pone.0006918 (PMC2735033; doi:10.1371/journal.pone.0006918)
Supplement: Methods S1 — (0.02 MB DOC) [file pone.0006918.s006.doc]

**Supplementary Methods**

***In Situ* hybridization**

RNA *In situ* hybridization and immunohistochemistry were performed as previously described {Guo, 1999 #1055}.

**Morpholino and injection**

*CDK9* Morpholino (MO) antisense oligonucleotide (Gene Tools, Corvallis OR) was designed to complement the exon 2/intron 2 junction. The MO sequence was: ACATCAAATACTCACCCAAAGGTGC. 1-2 nl of the morpholino oligonucleotide was injected at a concentration of 1.25mM.

**Flavopiridol treatment**

The *Nspt5*-injected embryos were transferred to the glass tubes with 0µM, 0.2µM and 1µM Flavopiridol and incubated from ~8-cell stage to 4-somite stage. The embryos were subsequently collected for RNA extraction and real-time PCR analysis. Differential interference contrast microscopy was performed on a Zeiss Axiophot 2 microscope for the embryos that were treated by 0µM and 1µM Flavopiridol.
